# Constrictive pericarditis heart failure in a patient with atrial fibrillation: A diagnostic challenge

**DOI:** 10.1002/ccr3.7166

**Published:** 2023-05-03

**Authors:** Rebeca Muñoz‐Rodríguez, Aída T. Igareta‐Herraiz, María Manuela Izquierdo‐Gómez, Belén Marí‐López, María Amelia Duque‐González, Flor Baeza‐Garzón, Antonio Barragán‐Acea, Julio Miranda‐Bacallado, Francisco Bosa‐Ojeda, Juan Lacalzada‐Almeida

**Affiliations:** ^1^ Department of Cardiology University Hospital of de Canary Islands Tenerife Spain

**Keywords:** atrial fibrillation, constrictive pericarditis, echocardiography

## Abstract

Constrictive pericarditis is an infrequent cause of heart failure. Diagnosis is challenging and requires a high level of suspicion. Subtle echocardiographic findings, as the pericardial bounce, could be the clue to diagnosis.

## Graphical abstract

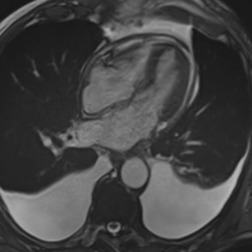

## 1 INTRODUCTION

We describe the case of a 86‐year‐old patient with a heart failure syndrome with preserved ejection fraction of unknown etiology and coexistence of atrial fibrillation in whom the presence of pericardial echocardiographic bounce was the clue to an accurate diagnosis and of constrictive pericarditis.

Constrictive pericarditis (CP) is a potentially treatable cause of heart failure with preserved ejection fraction, in which a diseased and non‐compliant pericardium generates a reduction in the ventricular filling and, consequently, predominant right heart failure signs. Characteristic physical findings are increased jugular venous pressure with rapid “y” descent and Kussmaul's sign, edema, hepatomegaly, pericardial knock, and ascites. Despite the advances in cardiovascular imaging, the diagnosis of CP is usually challenging, requiring a high clinical suspicion and a multimodality diagnosis approach.^1^

Transthoracic echocardiography is the first‐line diagnostic test in patients presenting diastolic heart failure. However, the coexistence of atrial fibrillation prevents the accurate doppler velocity assessment which usually guides the diagnosis.^2^ Subtle echocardiographic findings, as the pericardial bounce, could be the clue for an appropriate diagnostic and therapeutic approach.

## 2 CASE PRESENTATION

A 86‐year‐old man was admitted at our Cardiology Unit (University Hospital of the Canary Islands, Tenerife) after 3 months of progressive NYHA III dyspnea, orthopnea, paroxysmal nocturnal dyspnea, and lower limb edema. The patient's medical history was notable for a type 2 diabetes mellitus no insulin‐dependent, arterial hypertension, dyslipidemia, and a chronic obstructive pulmonary disease related to a past smoking habit. In the last 2 years, the patient was suffering from a microcytic and iron deficient anemia, finding duodenal and cecal angiodysplasia.

On physical examination, the patient was afebrile with a blood pressure of 100/55 mmHg, a heart rate of 100 beats/min, and a basal tachypnea of 26 breaths/min. Cardiac tones were arrhythmic, with moderate intensity and a mild ejection murmur with preserved and attenuated second tone was appreciated in the aortic focus. He had bilateral pleural effusion, ascitic semiology, and engorged jugular veins on his neck reaching the mandibular arch with Kussmaul's sign. The 12 leads electrocardiogram showed atrial fibrillation, incomplete right branch block, and low voltages. Despite ambulatory management with oral diuretics, the congestive heart failure clinic did not improve.

The clinical presentation, a right‐sided predominant heart failure syndrome, with refractory congestion, atrial fibrillation, incomplete right branch block, attenuated cardiac tones at the auscultation, and preserved ejection fraction at the echocardiographic assessment was highly supportive of a restrictive cardiomyopathy issue, being the senile amyloidosis the most plausible diagnosis. Moreover, as a IgM lambda paraprotein component was casually found in the routine analysis, a light‐chain amyloidosis was suspected. Nevertheless, the patient did not show other systemic symptoms or signs associated with amyloidosis, as atraumatic rupture of the biceps or polyneuropathy.

The initial transthoracic echocardiogram showed a non‐dilated left ventricle with minimum hypertrophy and preserved ejection fraction. An abnormal ventricular septal motion, strongly suggestive of pericardial bounce, was objectified and ascertained, given the poor acoustic window, with contrast echo (Video S1). Dilated inferior cava vein with diastolic reversal flow was also found. The NTproBNP was 2448 pg/dL and a stage 3 acute kidney injury was objectified. A monoclonal IgM lambda paraprotein component was uncovered on the proteinogram. Mantoux test was negative. The thoracic X‐ray showed bilateral pleural effusion and no calcium was seen in the cardiac silhouette.

Spontaneous cardioversion to sinus rhythm occurred during admission. Transthoracic echocardiogram with a complete doppler evaluation was performed in sinus rhythm, showing a significant transmitral and transtricuspid flow variation with breathing (Figure 2) and an increased media mitral annular e′ velocity.

**FIGURE 1:**
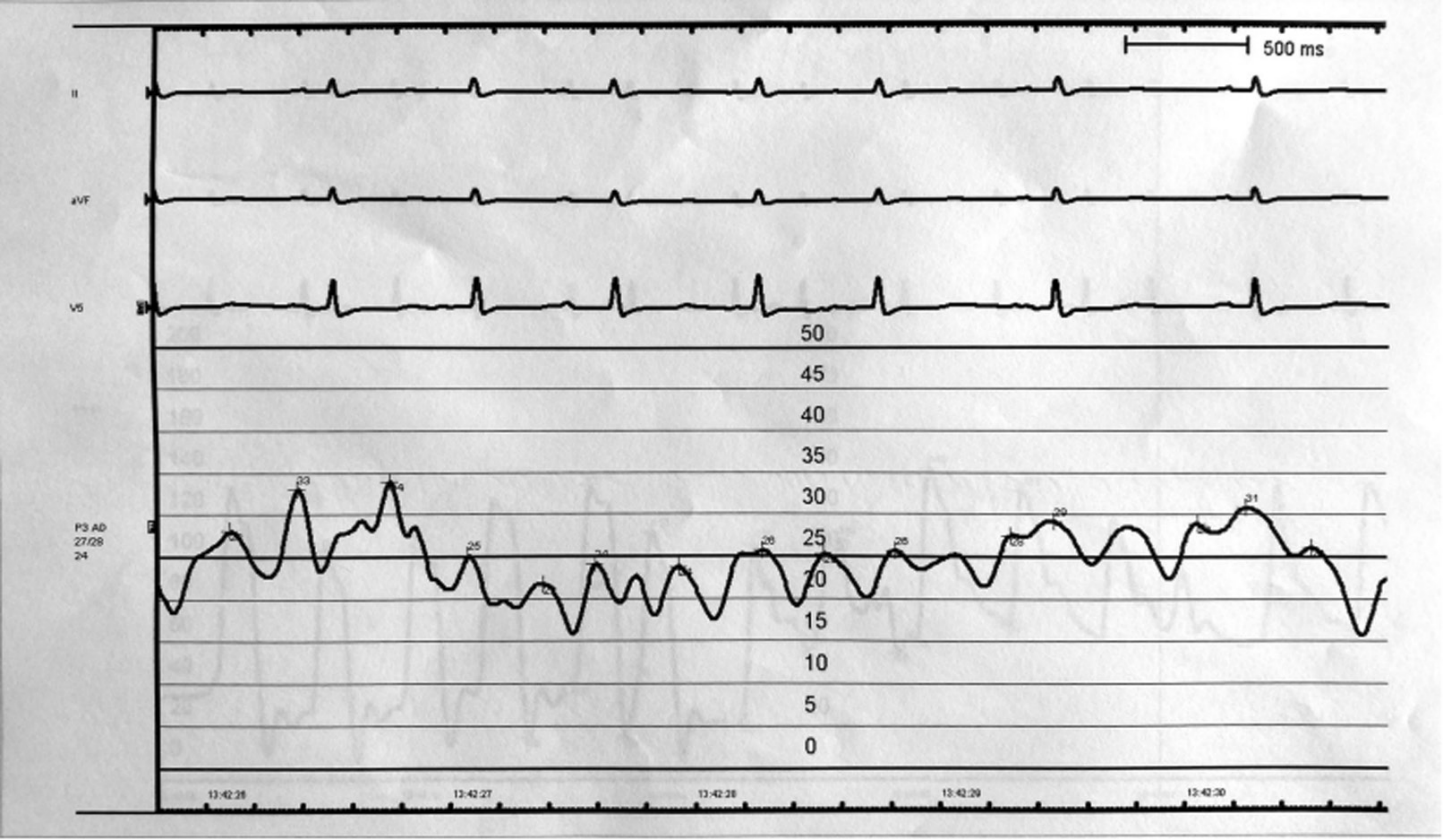
Right atria hemodynamic catheterization. Preserved *x* descent, prominent *y* descent and similar *a* and *v* wave values, strongly suggestive of constrictive pericarditis, can be observed.

A normal coronary angiography showed an absence of significant coronary lesions, and the invasive hemodynamic study demonstrated severe right‐sided heart failure. Both capillary pulmonary and right atrial pressure curves showed a constrictive pattern (Figure 1). A myocardial scintigraphy ruled out a transthyretin amyloidosis. A cardiac magnetic resonance evinced a mild anterior pericardial thickening of 4 mm with a pericardial bounce (Video S2). Native T1 mapping was normal. After multimodality assessment, the diagnosis of CP was established.

**FIGURE 2:**
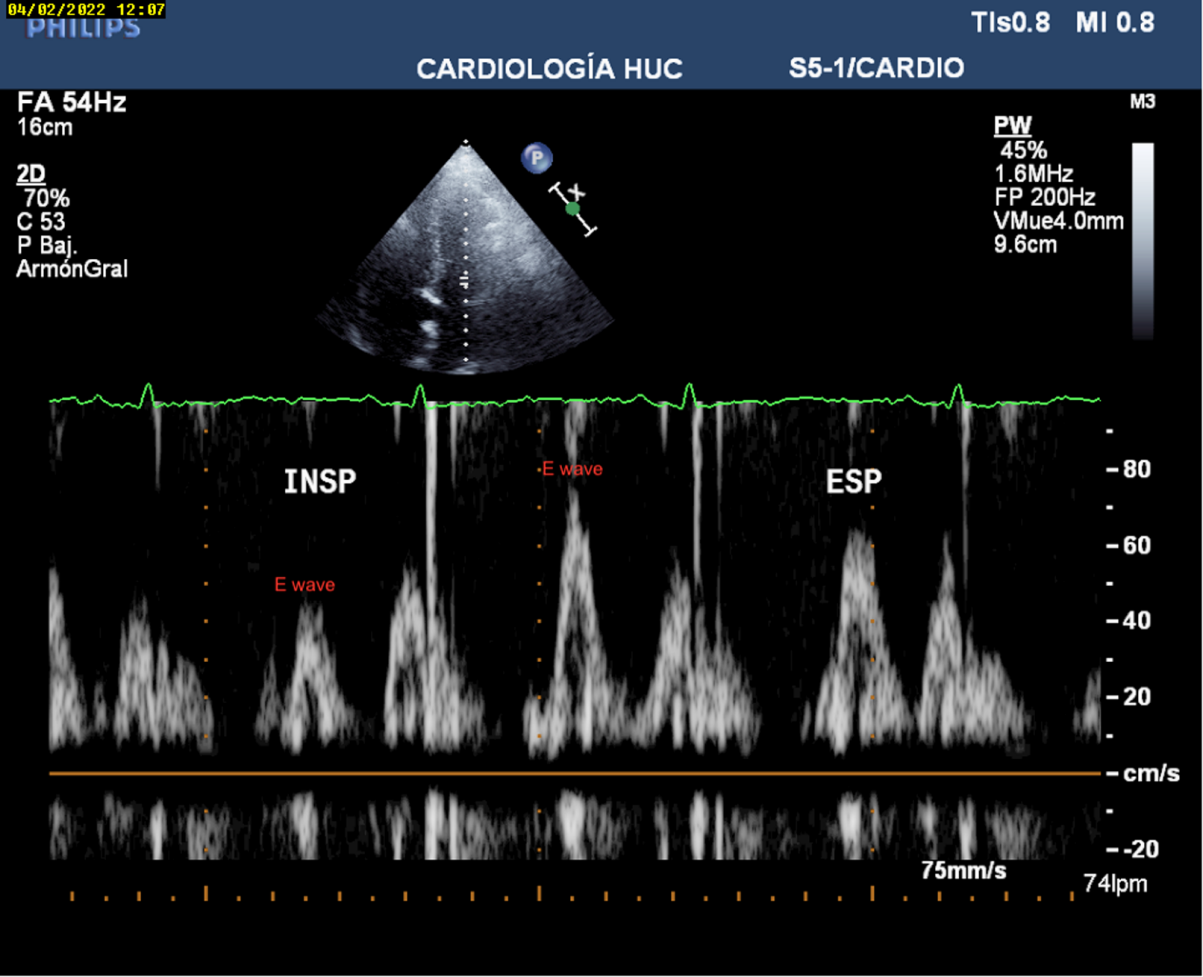
Transmitral doppler assessment. An E‐wave velocity decrease over 25% with inspiration can be observed.

The Hematology Unit was asked for an assessment due to the proteinogram findings, performing a narrow bone aspiration, and incidentally, a small lymphocyte B lymphoma, probably a splenic lymphoma of the marginal zone, was diagnosed.

This case was discussed in the weekly Heart Team session. In spite of being an elderly patient and the recent diagnosis of a lymphoma, he did not meet frailty criteria and his hematological pathology showed a good prognosis. While the reported studies were being completed, medical therapy with whole diuretic nephron block was established with scarce clinical improvement. The decision of a surgical approach was taken and a phrenic–phrenic pericardiectomy was performed.

The patient was discharged after 2 months of admission with a significant improvement of the heart failure signs. The pathological anatomy of the excised pericardium showed a chronic fibrosis with mononuclear inflammatory infiltration, suggestive of a chronic CP, without granulomas or calcium. The lymphoma did not show progression.

## 3 DISCUSSION

CP is a rare but potentially treatable cause of diastolic heart failure. Tuberculosis has been historically the most common etiology. Moreover, the development of a CP after an episode of acute pericarditis is seen in 1.8% of the patients.^2^ Its identification is challenging and may be difficult to make a precise differential diagnosis between other entities as restrictive cardiomyopathy, pulmonary hypertension, right‐sided ventricular infarction, or severe tricuspid regurgitation. A high level of clinical suspicion and a multimodality diagnosis approach with invasive hemodynamic assessment are required to confirm it.^3^

Transthoracic echocardiography is the first‐line diagnostic test in patients presenting diastolic heart failure. Even the pericardium evaluation is suboptimal with this technique, Doppler velocity assessment allows the study of the functional and hemodynamic consequences of the constricting pericardium.^4^ The American Society of Echocardiography recommends the M‐mode evaluation of the septal motion, the premature opening of the pulmonary valve and the inferior vena cava. Color M‐Mode could show an increased propagation velocity of early diastolic transmitral flow. Doppler evaluation must include assessment of fillings patterns of both left and right ventricle and its respiratory variation, as well as the tissue mitral annular doppler velocities.^4^

However, in 2014 in the Mayo Clinic, a blind fashion echocardiographic review of patients with surgically confirmed CP established three principal echocardiographic variables for clinical use: respiration‐related ventricular septal shift, media mitral annular e′ velocity, and the presence of hepatic vein expiratory diastolic reversal ratio. The presence of a combination of ventricular septal shift with either septal e′ of 9 cm/s or higher or hepatic expiratory diastolic reversal ratio higher than 0.79 showed the best combination of sensitivity (87%) and specificity (91%). The occurrence of the three criteria performs a specificity of 97%.^5^ In our case, the three criteria were met.

In this case, the coexistence of atrial fibrillation results in a more challenging diagnosis process. Around a 30% of patients with CP suffer from atrial fibrillation. In patients with CP, a prolonged disease duration and the presence of pericardial calcification are associated with a higher prevalence. The possibility of its development is a 27% by each year of disease duration.^6^ In this cases, the mitral inflow velocity and the pulmonary venous flow variation also show a significant variation when using transesophageal echocardiography.^7^ However, when the atrial arrhythmia prevents an accurate doppler assessment, the presence of solely one of the Mayo Clinic criteria has demonstrated a significant association with CP, notwithstanding the scarce cohort of patients with this pathology and atrial fibrillation or flutter in the literature.^5^ In addition, longitudinal and circumferential strain can be useful to differentiate between CP and restrictive cardiomyopathy.^4^

In conclusion, CP is an infrequent cause of preserved ejection fraction heart failure. Diagnosis is challenging and requires a high level of suspicion, even more when the patient is under atrial fibrillation. Subtle echocardiographic findings, as the pericardial bounce, could be the clue to an accurate multimodality diagnosis.

## AUTHOR CONTRIBUTIONS

**Rebeca Muñoz‐Rodríguez:** Conceptualization; data curation; investigation; project administration; writing – original draft. **Aida T. Tindaya:** Investigation; writing – original draft. **María Manuela Izquierdo‐Gómez:** Data curation; investigation. **Belén Marí‐López:** Data curation; investigation; resources. **María Amelia Duque‐Gonzalez:** Data curation; resources. **Flor Baeza‐Garzón:** Data curation; resources. **Antonio Barragán‐Acea:** Data curation; investigation; resources. **Julio Miranda‐Bacallado:** Conceptualization; data curation; resources. **Francisco Bosa‐Ojeda:** Conceptualization; supervision; validation. **Juan Lacalzada‐Almeida:** Conceptualization; resources; supervision; writing – review and editing.

## FUNDING INFORMATION

None declared.

## CONFLICT OF INTEREST STATEMENT

No conflicts of interest to declare.

5

## CONSENT

The data that support the findings of this study are available from the corresponding author upon reasonable request. Written informed consent was obtained from the patient to publish this report in accordance with the journal's patient consent policy.

## Supporting information


Video S1.

Video S2.

## Data Availability

The data that support the findings of this study are available from the corresponding author upon reasonable request. Written informed consent was obtained from the patient to publish this report in accordance with the journal's patient consent policy.
